# Vaccination against microbiota motility protects mice from the detrimental impact of dietary emulsifier consumption

**DOI:** 10.1371/journal.pbio.3002289

**Published:** 2023-09-19

**Authors:** Melissa C. Kordahi, Clara Delaroque, Marie-Florence Bredèche, Andrew T. Gewirtz, Benoit Chassaing

**Affiliations:** 1 INSERM U1016, Team “Mucosal microbiota in chronic inflammatory diseases”, CNRS UMR 8104, Université Paris Cité, Paris, France; 2 INSERM U1016, Team “Robustness and evolvability of life”, CNRS UMR 8104, Université Paris Cité, Paris, France; 3 Institute for Biomedical Sciences, Centre for Inflammation, Immunity and Infection, Digestive Disease Research Group, Georgia State University, Atlanta, Georgia, United States of America; UT Southwestern: The University of Texas Southwestern Medical Center, UNITED STATES

## Abstract

Dietary emulsifiers, including carboxymethylcellulose (CMC) and polysorbate 80 (P80), perturb gut microbiota composition and gene expression, resulting in a microbiota with enhanced capacity to activate host pro-inflammatory gene expression and invade the intestine’s inner mucus layer. Such microbiota alterations promote intestinal inflammation, which can have a variety of phenotypic consequences including increased adiposity. Bacterial flagellin is a key mediator of emulsifiers’ impact in that this molecule enables motility and is itself a pro-inflammatory agonist. Hence, we reasoned that training the adaptive mucosal immune system to exclude microbes that express flagellin might protect against emulsifiers. Investigating this notion found that immunizing mice with flagellin elicited an increase in mucosal anti-flagellin IgA and IgA-coated microbiota that would have otherwise developed in response to CMC and P80 consumption. Yet, eliciting these responses in advance via flagellin immunization prevented CMC/P80-induced increases in microbiota expression of pro-inflammatory agonists including LPS and flagellin. Furthermore, such immunization prevented CMC/P80-induced microbiota encroachment and deleterious pro-inflammatory consequences associated therewith, including colon shortening and increased adiposity. Hence, eliciting mucosal immune responses to pathobiont surface components, including flagellin, may be a means of combatting the array of inflammatory diseases that are promoted by emulsifiers and perhaps other modern microbiota stressors.

## Abstract 2

Bacterial flagellin is a key mediator of detrimental impacts on intestinal health caused by dietary emulsifier. This study shows that immunizing mice against flagellin can prevent emulsifier-induced changes in microbiota pro-inflammatory potential, microbiota encroachment, as well as deleterious consequences such as intestinal inflammation and metabolic deregulation.

## Introduction

The intestinal tract is colonized by a large and diverse collection of microbes referred to as the gut microbiota [1]. Under physiological conditions, the intestine is protected from its microbiota by a multilayered mucus structure that covers the intestinal surface, thus allowing the vast majority of gut bacteria to be kept at a safe distance from the intestinal epithelial lining [2]. We and others have reported that select dietary emulsifiers can promote low-grade inflammation (LGI) in the gut. Such LGI associates with, and may result from, emulsifiers changing gut microbiota. LGI can contribute to a variety of chronic diseases, including metabolic syndrome and can predispose to severe forms of inflammation including inflammatory bowel disease (IBD) [3–5]. We, for example, previously reported that 2 commonly used emulsifiers, namely, carboxymethylcellulose (CMC) and polysorbate 80 (P80), are sufficient to induce colitis in mice genetically prone to this disorder as well as to promote metabolic dysregulations in wild-type (WT) mice. Mechanistically, such alterations were associated with host–microbiota perturbations characterized by altered microbiota composition and function, especially the promotion of microbiota encroachment, hypothesized to be central for the observed host damages. Microbiota encroachment was previously reported to involve, at least in part, the flagella appendix expressed by select microbiota members [6–8] and responsible for bacterial motility [6,7]. We indeed previously observed a direct correlation between the severity of microbiota encroachment and the severity of emulsifiers-induced chronic intestinal inflammation in mice [3], as well as with the severity of type 2 diabetes in a human cohort [9]. Using gnotobiotic mouse models hosting a minimally complex intestinal microbiota, we observed that in the absence of microbiota encroachment, dietary emulsifiers are well tolerated and not associated with detrimental consequences on intestinal health. Altogether, these findings suggest that microbiota encroachment, likely mediated, at least in part, by flagella expression, is central for the subsequent development of chronic intestinal inflammation and metabolic dysregulations, in both preclinical models as well as in humans.

It has also been observed that levels of bacterial flagellin—the main component of bacterial flagella—are usually low in a healthy intestine and increased in an inflamed microenvironment, such as in IBD [10–12]. In mice lacking the flagellin receptor Toll-like receptor 5 (TLR5), a loss of flagellin-specific immunoglobulins (Igs) response is associated with an increased proportion of flagellated bacteria in the intestinal tract in a way that associates with microbiota encroachment [13]. Importantly, previous work demonstrating that anti-flagellin Igs can directly down-regulate/shutdown flagellar gene expression and motility by select bacteria suggest a direct relationship between intestinal anti-flagellin and microbiota-derived flagellin expression [14]. Well aligned with this concept, inducing an intestinal flagellin-specific IgA response decreased levels of flagellated bacteria, reducing microbiota encroachment, which altogether protects against experimentally induced severe and low-grade inflammation [6]. Interestingly, the beneficial impact of such anti-flagella adaptative immune response appears to be important only before disease initiation, with the observation that established chronic intestinal inflammation associates with nonprotective immune reactivity against flagella likely due to microbiota breaching the epithelial lining [15,16].

Based on these previous observations, we hypothesized here that inducing a mucosal flagellin-specific IgA response through purified flagellin immunization can prevent against dietary emulsifiers–induced detrimental consequences. We observed that flagellin immunization is sufficient to fully prevent emulsifiers-induced alterations in microbiota composition and localization. Furthermore, such immunization efficiently protected against dietary emulsifiers–induced low-grade intestinal inflammation and metabolic dysregulation. Hence, the protective potential of flagellin immunization supports the central role played by this bacterial appendix in promoting microbiota encroachment and downstream detrimental consequences in a way that can be used to combat modern dietary stressors known to perturb host–microbiota interactions.

## Results

### Flagellin immunization stabilizes IgA–microbiota interaction

**Fig 1:**
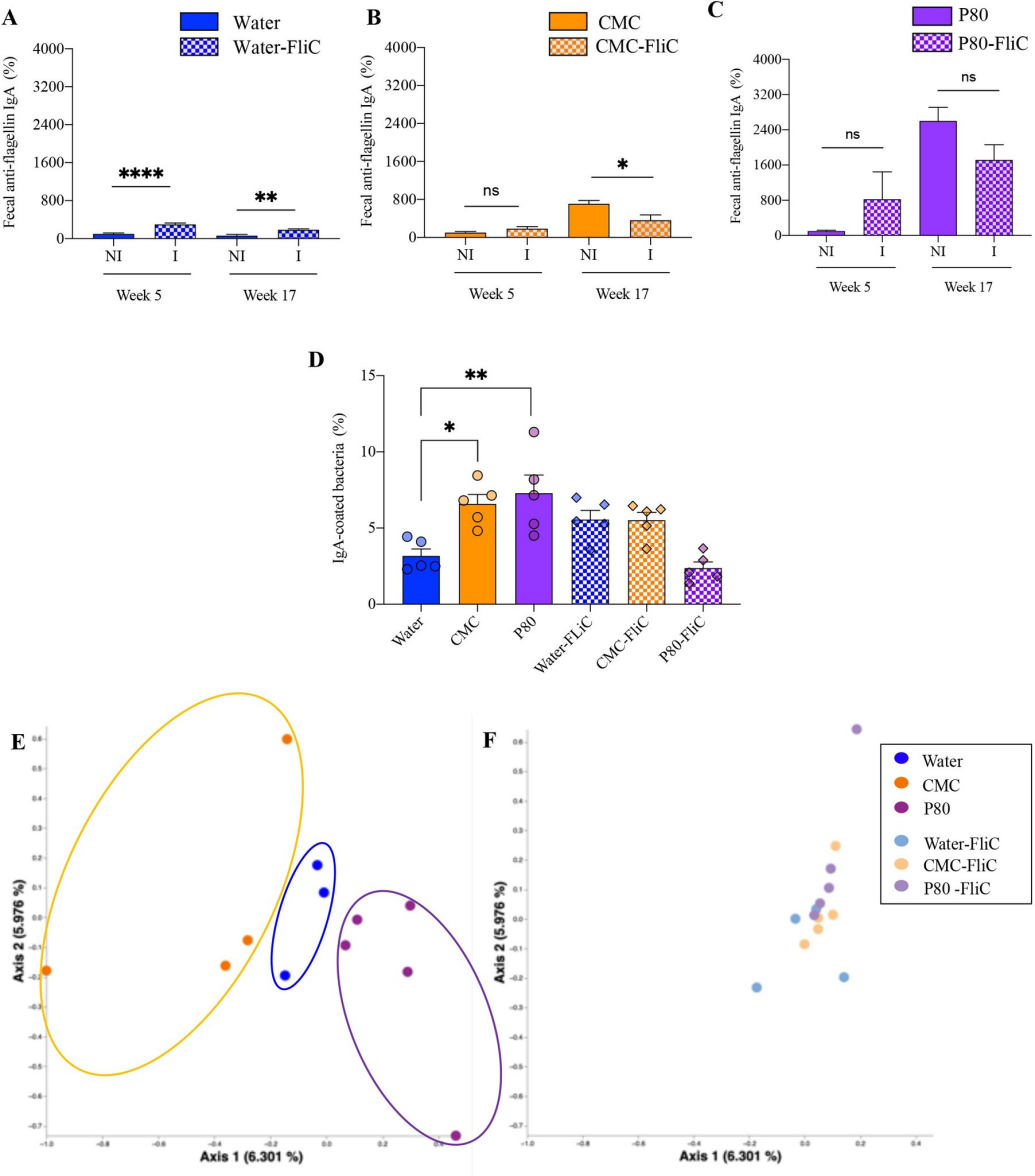
Flagellin immunization stabilizes IgA–microbiota interaction. (**A-C**) Fecal levels of anti-flagellin IgA at weeks 5 and 17, with data being expressed as relative values compared to week 5 nonimmunized group, defined as 100%. (**D-F**) Cecal contents, collected at week 19, were sorted for IgA-positive and IgA-negative bacterial populations. DNA was extracted from sorted cells and subjected to 16S rRNA sequencing. (**D**) Bar graph representing the percentage of IgA-coated bacteria in the caecum content at week 19. (**E, F**) PCoA of the Euclidean distance, at week 19, using IgA indices with dots being colored by treatment (**E**, water = blue; CMC = orange; P80 = purple, **F,** water-FliC = light blue; CMC-FliC = light orange; P80-FliC = light purple). The underlying data for this figure can be found in S1 Data. *N =* 4–5. For bar graphs, statistical analyses were performed using a *t* test and one-way ANOVA. For line charts, a two-way ANOVA or a mixed model was performed. Significant differences were recorded as follows: CMC vs. water, *p < 0.05, **p < 0.01, ****p < 0.0001. ANOVA, analysis of variance; CMC, carboxymethylcellulose; FliC, flagellin; IgA, immunoglobulin A; PCoA, principal coordinate analysis; P80, polysorbate 80.

We and others previously reported that chronic consumption of dietary emulsifiers can detrimentally impact host health by promoting chronic intestinal inflammation [4,5]. In order to test the potential beneficial effect of flagellin immunization, groups of mice were chronically exposed to emulsifiers CMC or P80 following a regimen of weekly immunization with purified bacterial flagellin (or PBS for the control groups) that lasted for 7 weeks prior to emulsifiers exposure, as schematically represented in **S1 Fig**. We first examined to which extent such immunization regimen impacts fecal IgA response. In accordance with previous studies [6], flagellin immunization was sufficient to increase levels of fecal anti-flagellin IgA (**Fig 1A–1C** and **S1 Data,** week 5), while, unsurprisingly, fecal anti-flagellin IgA levels were stable in nonimmunized water-treated mice (**Fig 1A and S1, S2 and S6 Data files**, week 17). Yet, nonimmunized mice exhibited increased levels of flagellin-specific IgA following prolonged emulsifiers consumption (**Fig 1B and 1C and S2, S1 and S6 Data files**, week 17). Such observation aligns with previous observations made in IBD patients, which harbor elevated immunoreactivity against bacterial flagellin, likely a consequence of increased exposure of microbiota to the underlying immune system. Similarly, dietary emulsifiers–induced chronic intestinal inflammation, which associates with elevated microbiota pro-inflammatory potential and microbiota encroachment, is ultimately leading to elevated immunoreactivity against bacterial flagellin (**Fig 1B and 1C and S1 Data**). Moreover, the flagellin immunization regimen used was sufficient to prevent such fecal anti-flagellin immunoreactivity, suggesting that the promotion of anti-flagellin antibody response prior to emulsifiers exposure prevented emulsifiers-induced anti-flagellin antibody response (**Fig 1B and 1C and S1 Data**). Analogous observations were made regarding total IgA-coated bacterial population, with emulsifiers consumption inducing a significant increase in IgA+ fecal bacterial populations in nonimmunized mice, while flagellin immunization partially prevented such emulsifiers-induced changes, particularly in the P80-consuming groups (**Fig 1D and S1 Data**). Altogether, these observations indicate that prechallenge immunization of the intestinal mucosa against bacterial antigen stabilizes microbiota–immune system interaction in a way that we hypothesize to be sufficient to prevent emulsifiers-induced chronic intestinal inflammation and downstream consequences.

We next performed cecal IgA+ and IgA− bacterial population sorting and 16S sequencing, as previously reported [6], in order to characterize the impact of flagellin immunization on emulsifiers-induced modulation of the IgA-coated microbial population. As presented in **S3 Fig** (**S7 Data**), such an approach showed a great range of IgA indices in all the tested experimental groups and without evident global effect of emulsifiers consumption nor flagellin immunization. Accordingly, principal coordinate analysis (PCoA) of Euclidean distances using computed IgA index (log (IgA^+^ abundance / IgA^−^ abundance)) revealed that both CMC and P80 consumption significantly impacted IgA coating of the intestinal microbiota, with clear distinct clustering between groups (**Fig 1E**). As presented in **S4 Fig** (**S8 Data**), both CMC and P80 consumption induced alterations in the IgA coating of numerous Clostridiales microbiota members. Such emulsifiers-induced alteration in the IgA-coated microbial population was mostly prevented by flagellin immunization, as indicated by the absence of treatment-based clustering (**Fig 1F**). Flagellin immunization impacted the IgA index from various members of the intestinal microbiota, including Lachnospiraceae, Ruminococcaceae, and Bacteroidaceae (**S5 Fig and S9 Data**). Thus, the impact of emulsifiers consumption on the IgA–microbiota interaction was not observed in immunized mice, suggesting that training the immune system to target flagellin prevented these compounds from destabilizing microbiota–immune system homeostasis.

### Flagellin immunization alters microbiota composition but does not prevent emulsifiers-induced microbiota alterations

**Fig 2:**
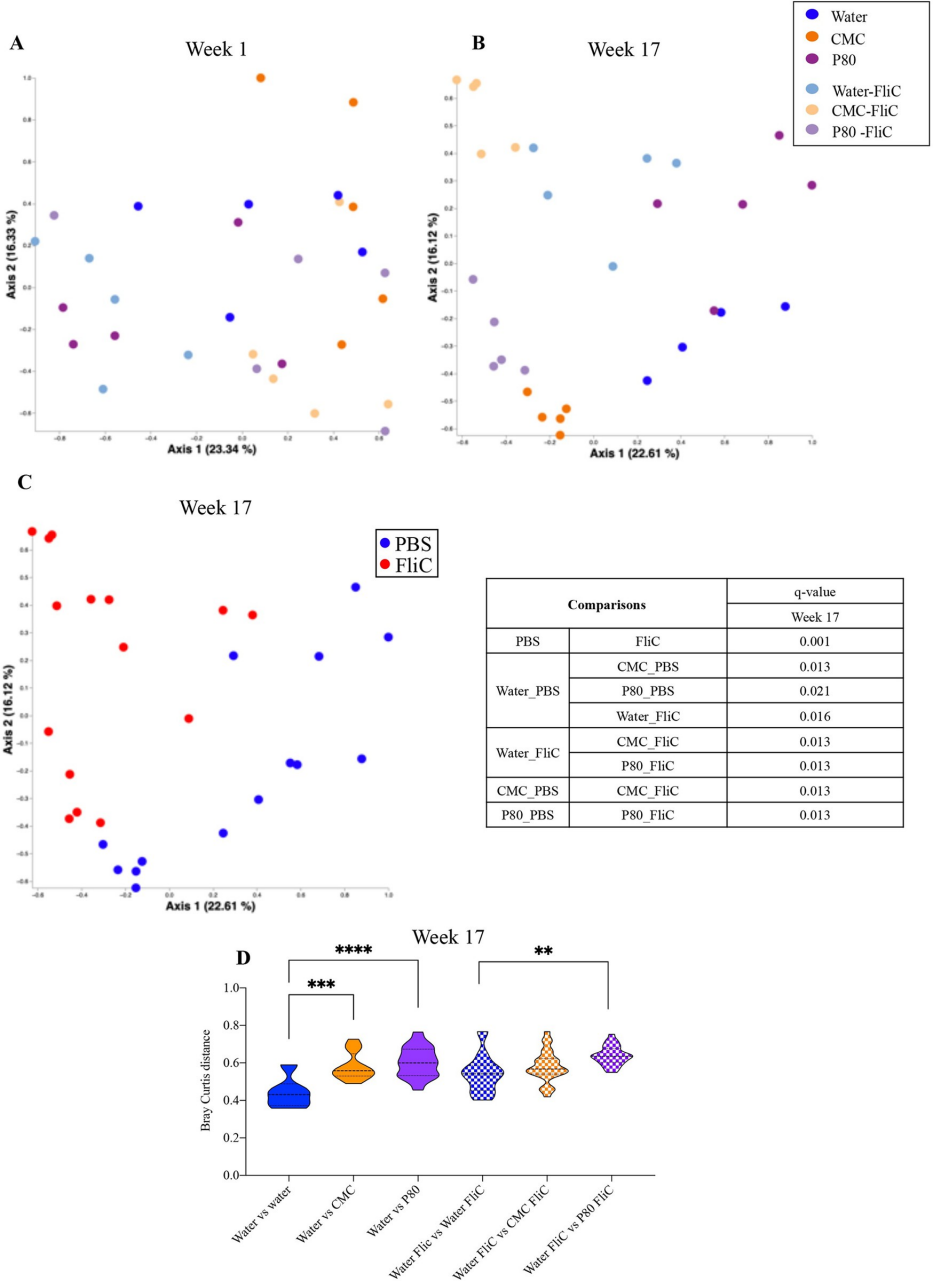
Flagellin immunization alters microbiota composition but does not prevent emulsifiers-induced alterations. Bacterial DNA was extracted from feces collected at weeks 1 and 17 and subjected to 16S rRNA gene sequencing. (**A-C**) PcoA of the Bray Curtis distance matrix of microbiota assessed by 16S rRNA gene sequencing at week 1 (**A**) and week 17 (**B** and **C**). Each dot represents an individual animal and is colored by experimental group (in **A** and **B**: blue, water; orange, CMC; purple, P80; light blue, water-FliC; light orange, CMC-FliC; light purple, P80-FliC. In **C**: blue, PBS control groups; red, flagellin-immunized groups). (**D**) Bray Curtis distance separating mice between experimental groups at week 17. The underlying data for this figure can be found in S2 Data. Data are represented as means ± SEM. Statistical analyses were performed using a one-way ANOVA, and significant differences were recorded as follows: **p < 0.01, ***p < 0.001, ****p < 0.0001. ANOVA, analysis of variance; CMC, carboxymethylcellulose; FliC, flagellin; PCoA, principal coordinate analysis; P80, polysorbate 80.

The direct impact of dietary emulsifiers on the intestinal microbiota plays a central role in promoting bacterial encroachment, intestinal inflammation, and its downstream consequences [3,4]. Hence, we next examined the extent to which flagellin immunization might prevent emulsifiers-induced alterations in intestinal microbiota composition. Use of 16S rRNA gene sequencing followed by PCoA of the Bray Curtis distance revealed that mice included in the study had homogeneous baseline microbiota composition prior to the start of treatment (week 1, **Fig 2A**). In contrast, such approach found that 10 weeks of exposure to CMC or P80 resulted in a clear treatment-based microbiota clustering (week 17, **Fig 2B**), indicating that both CMC and P80 markedly impacted intestinal microbiota composition. Such observation was confirmed through Bray Curtis distance computing between groups, revealing a significant increase between emulsifiers-treated and water-treated mice (**Fig 2D and S2 Data**). Next, we observed that flagellin immunization is by itself also sufficient to clearly impact microbiota composition, with a distinct clustering being observed between immunized and nonimmunized mice (**Fig 2C**), with a PERMANOVA *p*-value of 0.001. Such microbiota composition investigation finally revealed that flagellin immunization is not sufficient to prevent emulsifiers-induced alterations in microbiota composition, with the observation of distinct clusterings (**Fig 2B**) and a significantly increased Bray Curtis distance between water- and P80-treated mice (**Fig 2D and S2 Data**). Altogether, these findings indicate that while flagellin immunization is sufficient to impact intestinal microbiota composition, it fails to prevent emulsifiers-induced microbiota alteration, suggesting that protection conferred by immunization on intestinal inflammatory tone and metabolic dysregulations does not solely rely on microbiota composition normalization.

### Flagellin immunization inhibits emulsifiers-induced microbiota pro-inflammatory potential and encroachment

**Fig 3:**
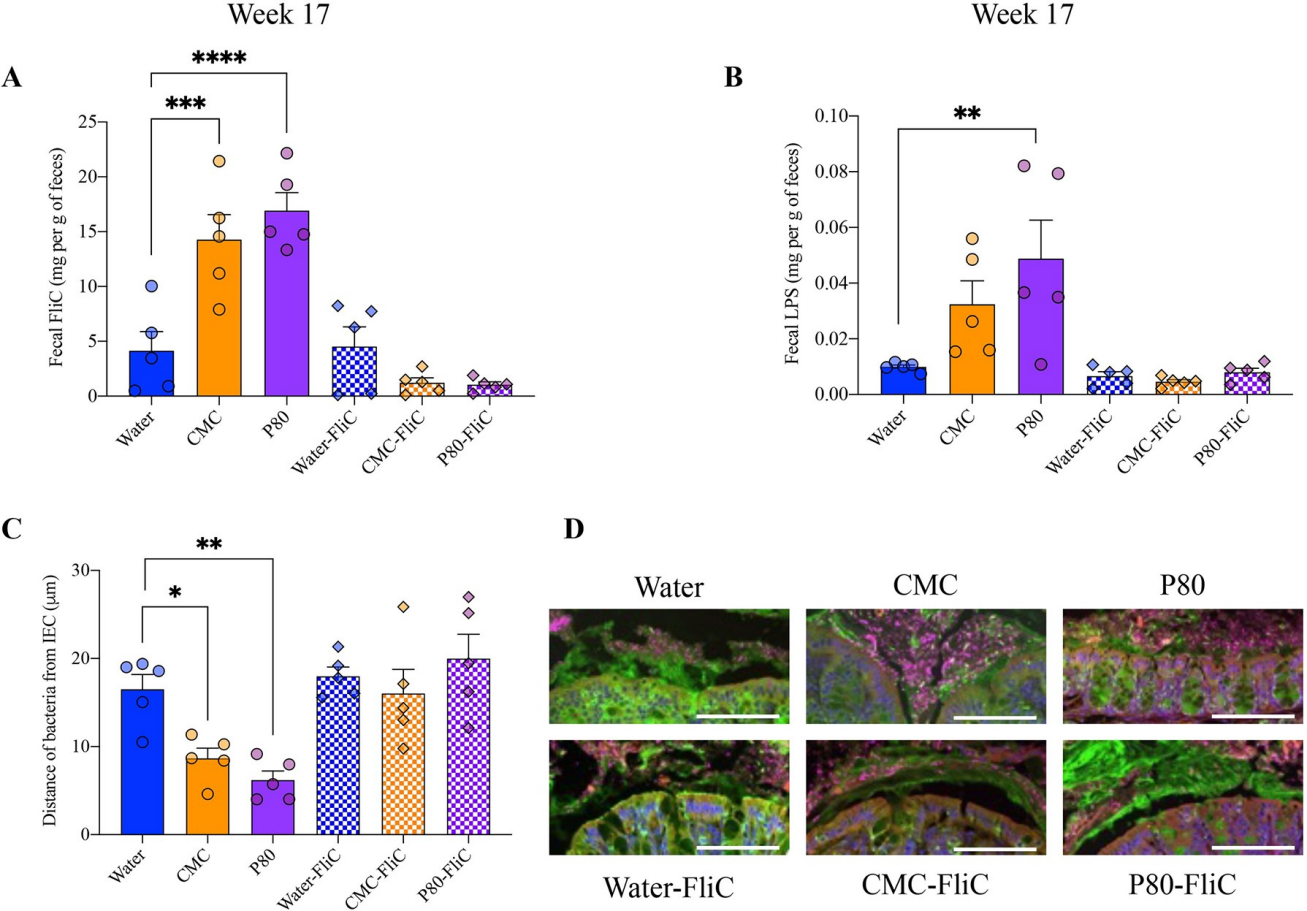
Flagellin immunization inhibits emulsifiers-induced microbiota pro-inflammatory potential and encroachment. (**A-C**) Fecal bioactive levels of pro-inflammatory microbiota-derived molecules flagellin (**A**) and LPS (**B**) measured at week 17 via use of TLR5 and TLR4 reporter cells. (**C**) Colons, collected at week 19, were subjected to immunostaining paired with FISH followed by confocal microscopy analysis of microbiota localization. Distances of closest bacteria to IECs per condition over 5 HPFs per mouse. (**D**) Representative pictures obtained from 5 biological replicates. MUC2, green; actin, purple; bacteria, red; and DNA, blue. Scale bar, 50 μm. The underlying data for this figure can be found in S3 Data. *N =* 4–5. Statistical analyses were performed using a one-way ANOVA and significant differences were recorded as follows: *p < 0.05, **p < 0.01, ***p < 0.001, ****p < 0.0001. ANOVA, analysis of variance; CMC, carboxymethylcellulose; FISH, fluorescent in situ hybridization; FliC, flagellin; HPF, high-powered field; IEC, intestinal epithelial cell; LPS, lipopolysaccharide; P80, polysorbate 80.

Beside microbiota composition, functional assessment appears warranted to deeply investigate microbiota alterations with potential downstream detrimental consequences on intestinal health. For example, alterations in microbiota—including those induced by CMC and P80—can increase the levels of pro-inflammatory microbiota-derived molecules such as flagellin and lipopolysaccharide (LPS) [3,4,17]. Thus, we next quantified fecal bioactive levels of these pro-inflammatory molecules via the use of TLR5 and TLR4 reporter cells. This approach revealed significantly elevated flagellin and LPS levels in mice consuming emulsifiers (**Fig 3A and 3B and S3 Data**). Animals that had been immunized against bacterial flagellin were fully protected against such emulsifiers-induced increase in microbiota pro-inflammatory potential, as presented in **Fig 3A and 3B** (**S3 Data**).

Another central detrimental impact of dietary emulsifiers consumption is their ability to induce microbiota encroachment, with the observation of microbiota colonization of the normally sterile inner mucus layer, which can be quantified by measuring epithelium–bacteria distance [9]. Such microbiota encroachment is hypothesized to play a central role in emulsifiers-induced chronic low-grade intestinal inflammation and metabolic dysregulations [3]. Based on the role played by flagella appendix in microbiota encroachment phenomenon [7,8], we examined microbiota encroachment via confocal imaging of Carnoy-fixed colon specimens. We recapitulated observations that both CMC and P80 consumption induce stark microbiota encroachment, with the average bacteria/epithelium distance being reduced from 16.50 μm in water-treated mice to 8.70 μm and 6.20 in CMC- and P80-treated mice, respectively (**Fig 3C and 3D and S3 Data**). Flagellin immunization fully protected against emulsifiers-induced microbiota encroachment, with bacteria/epithelium distances of 17.90 μm, 16.00 μm, and 19.90 μm being observed for water-, CMC-, and P80-treated groups, respectively (**Fig 3C and 3D and S3 Data**), further supporting the hypothesis that immune responses to flagellin protect against chronic intestinal inflammation.

### Flagellin immunization prevents emulsifiers-induced low-grade intestinal inflammation

**Fig 4:**
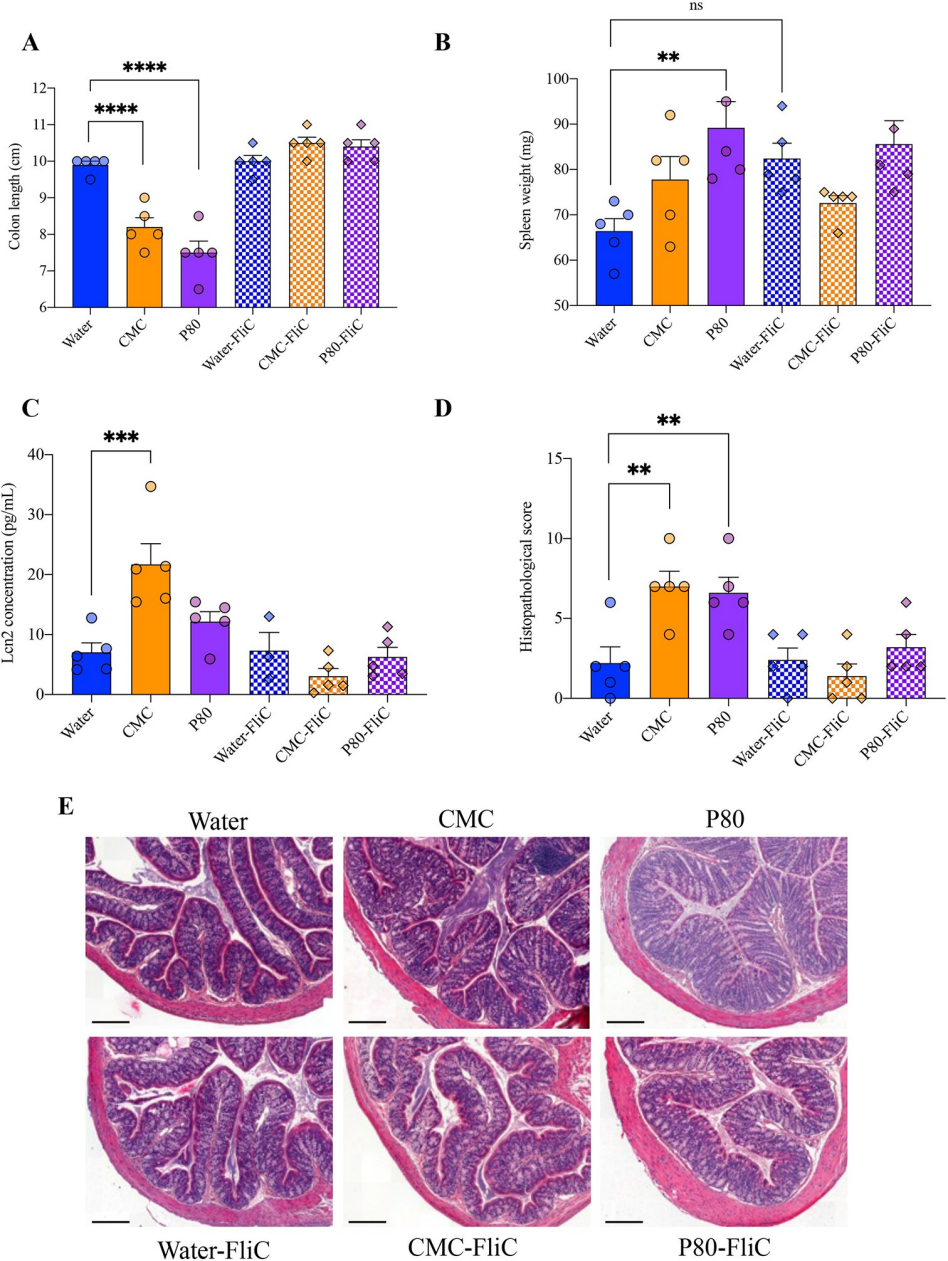
Flagellin immunization prevents emulsifiers-induced low-grade intestinal inflammation. (**A**) Colon length, (**B**) spleen weight, (**C**) fecal Lcn2 levels at euthanasia (week 19), (**D**) histopathological score of HE-stained colonic sections, and (**E**) representative HE-stained colonic sections. Scale bar, 100 μm. The underlying data for this figure can be found in S4 Data. Data are represented as means ± SEM. *N =* 4–5. Statistical analyses were performed using a one-way ANOVA and significant differences were recorded as follows: **p < 0.01, ***p < 0.001, ****p < 0.0001. ANOVA, analysis of variance; CMC, carboxymethylcellulose; FliC, flagellin; HE, hematoxylin–eosin; Lcn2, lipocalin-2; P80, polysorbate 80.

A central detrimental consequence of dietary emulsifiers consumption on hosts that consume them is the promotion of chronic intestinal inflammation [3,4]. As shown previously and herein, consumption of dietary emulsifiers induced chronic low-grade intestinal inflammation, as revealed by colon shortening, increase in spleen weight as well as in fecal levels of the pro-inflammatory marker Lipocalin-2 [18] (**Fig 4A–4C and S4 Data**) in emulsifiers-treated mice compared to water-treated control group. Colonic section, hematoxylin–eosin (HE) staining, and histopathological scoring confirmed the development of chronic low-grade intestinal inflammation in emulsifiers-treated mice with a score going from 2.20 ± 2.28 in water-treated mice to 7.00 ± 2.12 and 6.60 ± 2.19 in CMC- and P80-treated groups, respectively (**Fig 4D and 4E and S4 Data**). In contrast, mice immunized with flagellin lacked these indices of emulsifiers-induced chronic intestinal inflammation (**Fig 4 and S4 Data**). Indeed, histopathological score was, for example, entirely normalized, with values of 2.40 ± 1.67, 1.40 ± 1.67, and 3.20 ± 1.79 in water-, CMC-, and P80-treated groups, respectively (**Fig 4D and 4E and S4 Data**).

### Flagellin immunization dampens emulsifiers-induced metabolic dysregulations

**Fig 5:**
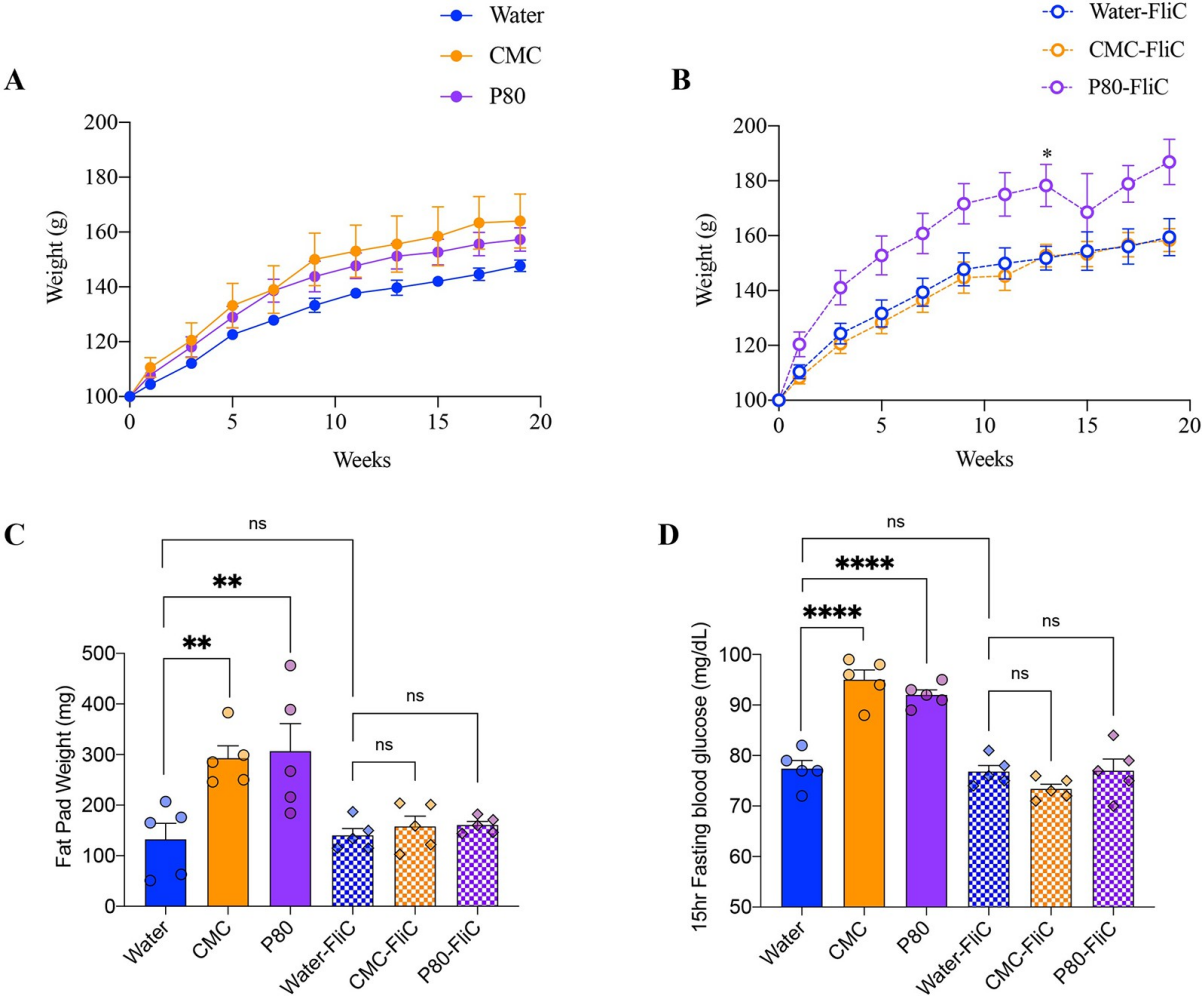
Flagellin immunization dampens emulsifiers-induced metabolic dysregulations. (**A, B**) Body weight gain over time of mice immunized with either vehicle (PBS, **A**) or purified flagellin (**B**). (**C**) Epididymal fat pad weight and (**D**) 15-hour fasting blood glucose level, measured at week 19. The underlying data for this figure can be found in S5 Data. Data are represented as means ± SEM. *N =* 4–5. For bar graphs, statistical analyses were performed using a one-way ANOVA and significant differences were recorded as follows: ns: non-significant, **p < 0.01, ****p < 0.0001. ANOVA, analysis of variance; CMC, carboxymethylcellulose; FliC, flagellin; P80, polysorbate 80.

We previously reported, in numerous models, that chronic intestinal inflammation can lead to metabolic dysregulations [3,17]. Hence, we next investigated the effect of dietary emulsifiers consumption on host metabolism, and the potential preventive effect of flagellin immunization. As reported in **Fig 5** (**S5 Data**), we observed that either CMC or P80 consumption induced a greater body weight gain compared with the water-treated control group (**Fig 5A and S5 Data**). Such increased body weight gain was accompanied by significantly increased fat deposition and overnight fasting blood glucose levels in emulsifiers-treated mice compared to control mice (**Fig 5C and 5D and S5 Data**), further demonstrating that chronic consumption of dietary emulsifiers is sufficient to impair host metabolism. Immunization with purified flagellin fully abrogated various emulsifiers-induced alterations in metabolism, with fat deposition and overnight fasting blood glucose levels being fully normalized to water-treated control group (**Fig 5C and 5D and S5 Data**). Regarding overall weight gain, flagellin immunization was sufficient to prevent CMC-induced increased weight gain. Altogether, these data indicate that flagellin immunization prevents the chronic intestinal inflammation and improves some of its associated metabolic consequences that were otherwise observed in mice chronically exposed to dietary emulsifiers.

## Discussion

Microbiota dysbiosis is thought to play a central role in driving intestinal inflammation and, consequently, numerous chronic diseases with an inflammatory component [1]. Features of microbiota dysbiosis include alterations in species composition with an enrichment in flagellated bacteria, which can, for example, result from increases in Gamma-Proteobacteria, including motile pathobiont *Escherichia coli* strains [19,20] but can also result from other classes of bacteria, especially Firmicutes, up-regulating motility-related gene expression [14]. These disease-associated microbiotas expressing high levels of flagellin are also characterized by an increased capacity to penetrate the normally sterile inner mucus layer, a feature referred to as microbial encroachment. Such encroaching microbiotas are thought to play a significant role in driving gut inflammation, with, for example, the previous observation of a positive correlation between microbiota encroachment and the severity of intestinal inflammation in mice models as well as with the severity of metabolic dysregulation in humans [3,21]. While there is likely a broad array of underlying factors inducing microbiota dysbiosis and encroachment, various evidence supports a major role for environmental (i.e., nongenetic) determinants. For instance, we and others have previously shown that consumption of dietary emulsifiers can induce altered microbiota composition and encroachment, resulting in colitis in genetically susceptible mice and in LGI and metabolic syndrome in WT mice [3,4].

In this study, we importantly report that flagellin immunization is sufficient to prevent numerous detrimental consequences normally induced by dietary emulsifiers consumption, while such immunization did not appear sufficient to fully prevent emulsifiers-induced compositional changes in the microbiota. Hence, what we believe to be important regarding immunization-induced microbiota modulation is related to functional aspects, including microbiota localization and pro-inflammatory potential, rather than compositional aspects. Moreover, recent work from Clasen and colleagues report a high heterogeneity in the ability of a given flagellin to activate the TLR5 receptor, suggesting that a given microbiota could have a relatively high proportion of microbiota members expressing flagellin and yet have a weak TLR5 activation potential [22]. Hence, precise identification of the impact of the immunization protocol on microbiota composition, and more specifically on the flagellated bacterial population, will require follow-up studies. Future work will, for example, involve laser-capture microdissection-based collection of the inner intestinal mucus layer, in order to focus on mucus-associated microbiota, followed by shotgun metagenomics and identification of the flagellated bacterial population. In the current study, together with a decrease in flagellin bioactive levels in immunized mice, we also observed a decrease in fecal bioactive LPS levels, and the exact underlying mechanism will also deserve further investigations.

While the intestinal tract possesses various innate and adaptive immune mechanisms to keep the inner mucus layer sterile, for example, through the secretion of various antimicrobial peptides, our group has previously indicated that adaptive immunity, in particular mucosal production of flagellin-specific IgA, plays a key role in keeping motile bacteria in check [6]. We have indeed observed that eliciting anti-flagellin antibodies via immunization is an efficient strategy to protect against colitis as well as against diet-induced obesity [6]. Although antibodies directed against bacterial flagella could be highly species specific, many anti-flagellin antibodies can recognize highly conserved flagellin epitopes, such that inoculation of mice with Salmonella-derived flagellin generate antibodies that exhibit considerable cross-reactivity with other flagellins such as Clostridia flagellin [23]. Moreover, we have previously reported that flagellin derived from various microbiota members can bind TLR5 receptor as well as be recognized by antibodies raised against recombinant flagellin peptides from Firmicute *Roseburia hominis* [14]. However, the cross-reactivity of Salmonella-derived flagellin antibodies against various microbiota members, for example, Bacteroidetes species, deserves further investigations. Hence, even if the select microbiota members encroaching upon the epithelium upon dietary emulsifiers consumption have not yet been identified, and regardless of the fact that they will likely be different in various hosts, we hypothesize that eliciting a robust anti-flagellin response will nonetheless provide a degree of protection against the deleterious effects of chronic exposure to dietary emulsifiers.

We specifically observed that while fecal flagellin levels were increased by emulsifiers exposure, immunization with purified flagellin fully prevented such effect. More importantly, the impact of both CMC and P80 on microbiota localization, fecal IgA response, intestinal inflammatory tone, and metabolism was all prevented in immunized mice. Thus, flagellin immunization appears as an efficient way to prevent the detrimental consequences of emulsifiers consumption. A possible explanation for such observations is that the primary mechanism of action for flagellin immunization is through the stabilization of the anti-flagellin IgA response normally produced while consuming emulsifiers, hence prohibiting the microbiota from penetrating the inner mucus layer coating the colon and activating pro-inflammatory genes. Moreover, while our IgA-Seq approach performed herein clearly suggests that flagellin immunization is sufficient to prevent emulsifiers-induced alteration in the IgA-coated microbiota composition, we do not know yet if this is occurring through modulation of the global microbiota composition or through targeted modulation of flagellin expression by various microbiota members.

Previous quantitative reverse transcription PCR (qRT-PCR)–based analysis in the colon of mice consuming dietary emulsifiers revealed only subtle modifications of select cytokines, suggesting the induction of only low-grade chronic intestinal inflammation. We suspect that only few intestinal immune cell populations are impacted, in the gastrointestinal tract, by dietary emulsifiers consumption. Hence, future work will involve performing single-cell RNA-seq analysis in order to finely characterize emulsifiers-induced alteration of the intestinal immune landscape. Moreover, intraperitoneal injection of flagellin also leads to pro-inflammatory response through activation of the innate immune system via TLR5 and/or NLRC4 in the peritoneum but also likely systemically, which could play a role in the observed prevention of detrimental phenotypes following administration of emulsifiers. Of note, we previously reported that in μMT mice unable to produce antibodies owing to their lack of mature B cells, flagellin immunization regimen no longer results in the beneficial modulation of the intestinal microbiota, hence arguing that a significant portion of flagellin immunization’s impact on the microbiota is mediated by anti-flagellin antibodies [6]. While we anticipate that the same observation will hold true concerning the protection of emulsifiers-treated mice, such aspect will deserve further investigations. Moreover, irrespective of the exact underling mechanism, more targeted immunization of the intestinal mucosa, for example, through targeted delivery of recombinant flagellin, should now be deployed.

To conclude, our data presented here suggest that antibacterial immunization could be an efficient way to prevent microbiota encroachment in a way that will subsequently prevent the development of chronic debilitating diseases. Of note, the regimen used herein, with repeated injection of purified flagellin, is not applicable in clinical settings. However, based on the presence of basal levels of anti-flagellin antibodies in humans, we speculate that humans might exhibit a memory-type response to exogenously administered flagellin, which will likely make targeted mucosal immunization with recombinant flagellin effective [24,25]. Additionally, as flagellin immunization was observed to be sufficient to protect against subsequently administered dietary emulsifiers, we have yet to investigate the therapeutic potential of such immunization regimen in established chronic low-grade intestinal inflammation. However, our results herein suggest that increased fecal flagellin in response to emulsifiers consumption in nonimmunized mice is also associated with an increase in anti-flagellin IgA (**Fig S2 and S6 Data**), which does not appear to be sufficient to prevent the deleterious effects induced by emulsifiers consumption. Similarly, Crohn’s disease patients harbor high levels of anti-flagellin Igs, which are not sufficient to be protective [26], suggesting that anti-flagellin Igs might be protective only when elicited prophylactically.

The various points raised above highlight the need for extensive preclinical development in order to harness the use of microbiota-derived antigens to vaccinate against modern chronic diseases involving alterations in the intestine–microbiota interaction. Nonetheless, our results suggest that this approach has high potential to prevent chronic inflammatory diseases. Should the elicitation of flagellin-specific mucosal antibodies keep motile bacteria in check, prevent microbiota encroachment, and result in a generally less pro-inflammatory microbiota in humans, we submit that this approach might be an innovative prophylactic/therapeutic venue for the protection against a broad array of inflammatory diseases including IBD and metabolic syndrome.

## Materials and methods

### Ethics statement

Mice were housed at Georgia State University (Atlanta, Georgia, USA) under institutionally approved protocols (Institutional Animal Care and Use Committee (IACUC) # A18006). Georgia State’s Animal Welfare Assurance number in accordance with the Public Health Service (PHS) Policy for Humane Care and Use of Laboratory Animals is D16-00527 (A3914-01).

### Materials

Sodium CMC (average MW~250,000) and P80 were purchased from Sigma (Sigma, St. Louis, Missouri).

### Mice and flagellin immunization

Monomeric flagellin was purified from flagella isolated from *Salmonella Typhimurium* (SL3201, fljB−) via high-performance liquid chromatography (HPLC), and purity was verified as previously described [27,28]. C57BL/6 male mice WT were maintained at Georgia State University, Atlanta, Georgia, USA, under institutionally approved protocols (IACUC # A14033 and A18006). Mice were immunized with *Salmonella Typhimurium*–derived flagellin (10 μg; described above) through intraperitoneal injections weekly for a total of 7 injections, with control mice being administered vehicle (PBS). Mice were littermates and group housed. Mice were killed by CO_2_ inhalation, and colon length, colon weight, spleen weight, and adipose weight were measured. Serum, feces, organs, as well as intestinal contents from the cecum were collected for downstream analysis.

### Emulsifier agent treatment

Mice were exposed to CMC or P80 diluted in the drinking water (1.0%) (not blinded). The same water (reverse osmosis–treated Atlanta city water) was used for the water-treated (control) group, and all these solutions were changed every week. Body weights were measured weekly and are expressed as % compare to the initial body weight (day 0) defined as 100%. Fresh feces were collected weekly for downstream analysis.

### Overnight fasting blood glucose measurement

Mice were placed in a clean cage and fasted for 15 h. Blood glucose concentration was then determined using a Nova Max Plus Glucose Meter and expressed in mg/dL.

### Fecal lipocalin-2 quantification

As previously described [18], frozen fecal samples were reconstituted in PBS containing 0.1% Tween 20 at 100 mg/ml and vortexed for 20 min. The homogenate was then centrifuged at 12,000 rpm for 10 min at 4°C. Clear supernatants were collected and stored at − 20°C until analysis. Lcn2 levels were measured in the supernatants using DuoSet Murine Lcn2 ELISA kit (R&D Systems, DY1857).

### Fecal flagellin and lipopolysaccharide (LPS) load quantification

Quantification of flagellin and lipopolysaccharide was previously described using human embryonic kidney (HEK)-Blue-mTLR5 and HEK-Blue-mTLR4 cells, respectively (Invivogen, hkb-mtlr5 and hkb-mtlr4, respectively) [3,4]. Fecal material was resuspended in PBS to a final concentration of 100 mg/mL and homogenized for 10 s using a Mini-Beadbeater-24 without the addition of beads to avoid bacteria disruption. Samples were then centrifuged at 8,000 × *g* for 2 min, serially diluted the resulting supernatant, and applied to mammalian cells. Purified *E*. *coli* flagellin and LPS (Sigma, L2887) were used for standard curve determination using HEK-BluemTLR5 and HEK-Blue-mTLR4 cells, respectively.

### Fecal sample preparation for immunoglobulin quantification

Fecal sample collection from mice occurred up to 3 mo after the final flagellin administration. Sample preparation for ELISA has been previously described [29]. In brief, 100 mg of fecal pellets were homogenized in 3 mL of collection media consisting of 0.05 mg soybean trypsin inhibitor per ml of a 3:1 mixture of 1× PBS and 0.1 M EDTA (pH 7.4). Following centrifugation at 1,800 rpm for 10 min, the supernatant was centrifuged again at 14,000 rpm for 15 min at 4°C, and final supernatant was collected and stored with 20% glycerol and 2 mM phenylmethylsulphonyl fluoride (Sigma, P-7626) at − 20°C until analysis.

### Fecal anti-flagellin IgA/IgG

Quantification of anti-flagellin- specific IgA and IgG has been previously described [24–26]. In brief, 96-well microtiter plates (Costar, Corning, New York) were coated with 100 ng/well of laboratory-made *Salmonella Typhimurium*–derived in 9.6 pH bicarbonate buffer overnight at 4°C. Fecal samples from mice were then applied at a 1:4 dilution for 1 h at 37°C. After incubation and washing, the wells were incubated with either horseradish peroxidase–linked anti-mouse IgG (GE Healthcare Life Sciences, NA931V) or horseradish peroxidase–linked anti-mouse IgA (Southern Biotech, 1040–05). Quantification of Ig was then developed by the addition of 3,3′,5,5′-Tetramethylbenzidine, and the optical density was calculated by the difference between readings at 450 nm and 540 nm.

### Isolation of IgA-coated bacteria

IgA-coated bacteria were isolated and sequenced as previously described [30,31]. Briefly, frozen cecal content samples were thoroughly homogenized in PBS to a final concentration of 20 mg/mL. Cecal suspensions were centrifuged at 50*g*, for 15 min at 4°C, then filtered through a 40-μm sterile nylon mesh. Around 50 μL of the filtered suspension was then resuspended in 1 mL PBS and centrifuged at 8,000 × *g*, for 5 min at 4°C. Resulting bacterial pellets were resuspended in 100 μl blocking buffer (staining buffer containing 20% filtered normal rat serum (NRS)) and incubated for 20 min at 4°C before being washed with 100 1 mL of staining buffer (PBS containing 1% (w/v) NRS, filtered). Cells were then stained with 100 μl of staining buffer containing PE-conjugated anti-mouse rat IgA (1:12.5; eBioscience, 12–4204–82) for 25 min at room temperature away from light. Following 3 washes with staining buffer, pellets were resuspended in 1 mL of staining buffer. Data acquisition was performed on a Biorad S3 sorter. Samples were gated on appropriate side scattered light/forward scatter (SSC-A/FSC-A) gates prior to being selected for events. For each sample, 500,000 events were collected from the IgA^−^, IgA^+^, as well as from the total population into sterile tubes. Each fraction was stored at − 20°C prior to DNA extraction and sequencing of bacterial 16 S rRNA genes, as described below. IgA index were computed according to the following formula: log (IgA + taxon abundance / IgA–taxon abundance) [32].

### Bacterial DNA extraction

DNA was extracted from fecal and cell sorted samples using a QIAmp Fast Stool DNA kit (Qiagen Laboratories) with chemical disruption (Lyzing buffer).

### Microbiota analysis through 16S rRNA gene sequencing

16S rRNA gene amplification and sequencing were performed using the Illumina MISeq technology following the protocol of the Earth Microbiome Project (www.earthmicrobiome.org/emp-standardprotocols) with some slight modifications. The 16S rRNA genes, region V4, were PCR amplified from each sample using a composite forward and reverse primer containing a unique 12-base barcode, designed with the Golay error-correcting scheme used to tag PCR products from respective samples. The forward primer 515F was used 5′-*AATGATACGGCGACCACCGAGATCTACACGCT*XXXXXXXXXXXXTATGGTAATTGTGTGYCAGCMGCCGCGGTAA-3′: the italicized sequence is the 5′ Illumina adapter, the 12X sequence is the Golay barcode and the bold sequence is the primer pad, the italicized bold sequence is the primer linker and the underlined sequence is the conserved bacterial primer 515F. The 806R primer used was 5′-*CAAGCAGAAGACGGCATACGAGAT*AGTCAGCCAGCCGGACTACNVGGGTWTCTAA T-3′: the italicized sequence is the 3′ reverse complement sequence of Illumina adapter, the bold sequence is the primer pad, the italicized and bold sequence is the primer linker, and the underlined sequence is the conserved bacterial primer 806R. PCR reactions consisted of 5PRIME HotMasterMix (Quantabio, Beverly, Massachusetts, USA) 0.2 μM of each primer, 10 to 100 ng template, and reaction conditions were set as follow: 3 min at 95°C, followed by 30 cycles of 45 s at 95°C, 60 s at 50°C, and 90 s at 72°C on a Bio-Rad thermocycler. PCR products were then visualized by gel electrophoresis and purified with Ampure magnetic purification beads (Agencourt, Brea, California, USA). The products were then quantified using Quanti-iT PicoGreen dsDNA assay), and a master DNA pool was generated from the purified products in equimolar ratios. The pooled product was also quantified with the Quanti-iT PicoGreen dsDNA assay, followed by sequencing using an Illumina MiSeq sequencer (pair-end reads, 2 × 250 bp).

### 16S rRNA gene sequence analysis

QIIME2-version 2019 was used to analyze 16s rRNA sequences. These sequences were demultiplexed and quality filtered using Dada2 method with QIIME2 default parameters to detect and correct Illumina amplicon sequence data, generating a table of Qiime 2 artifact. Then, a tree was generated using the align-to-tree-mafft-fasttree command for phylogenetic diversity analysis, and we computed alpha and beta diversity analyses using the core-metrics-phylogenetic command. PCoA plots were used to assess variations between experimental groups (beta diversity). Unprocessed sequencing data are deposited in the European Nucleotide Archive under accession number PRJEB64212, publicly accessible at https://www.ebi.ac.uk/ena/browser/home.

### Hematoxylin–eosin staining and histopathologic analysis

Following euthanasia, colons (proximal colon, 2 first cm from the cecum) were placed in methanol-Carnoy’s fixative solution (60% methanol, 30% chloroform, 10% glacial acetic acid). Tissues were then washed in methanol 2 × 30 min, ethanol 2 × 15 min, ethanol/xylene (1:1) 15 min, and xylene 2 × 15 min, followed by embedding in paraffin with a vertical orientation. Tissues were sectioned at 5-μm thickness and stained with HE using standard protocols. HE-stained slides were assigned 4 scores based on the degree of epithelial damage and inflammatory infiltrate in the mucosa, submucosa, and muscularis/serosa, as previously described [18]. The 4 individual scores per colon were added, resulting in a total scoring range of 0 to 36 per mouse. Representative images were selected.

### Immunostaining of mucins and localization of bacteria by FISH

Mucus immunostaining was paired with fluorescent in situ hybridization (FISH), as previously described [33] in order to analyze bacteria localization at the surface of the intestinal mucosa [3,17]. In brief, colonic tissues (proximal colon, second cm from the cecum, without fecal material) were placed in methanol-Carnoy’s fixative solution (60% methanol, 30% chloroform, 10% glacial acetic acid) for a minimum of 3 h at room temperature. Tissues were then washed in methanol 2 × 30 min, ethanol 2 × 15 min, ethanol/xylene (1:1) 15 min, and xylene 2 × 15 min, followed by embedding in paraffin with a vertical orientation. Then, 5-μm sections were performed and dewaxed by preheating at 60°C for 10 min, followed by xylene 60°C for 10 min, xylene for 10 min, and 99.5% ethanol for 10 min. Hybridization step was performed at 50°C overnight with EUB338 probe (5′-GCTGCCTCCCGTAGGAGT-3′, with a 5′ labeling using Alexa 647) diluted to a final concentration of 10 μg/mL in hybridization buffer (20 mM Tris–HCl (pH 7.4), 0.9 M NaCl, 0.1% SDS, 20% formamide). After washing 10 min in wash buffer (20 mM Tris–HCl (pH 7.4), 0.9 M NaCl) and 3 × 10 min formamide in PBS, PAP pen (Sigma-Aldrich, Z377821) was used to mark around the section, and block solution (5% fetal bovine serum in PBS) was added for 30 min at 4°C. Mucin-2 primary antibody (rabbit H-300; Santa Cruz Biotechnology, sc-15334) was diluted 1:1,500 in block solution and apply overnight at 4°C. After washing 3 × 10 min in PBS, block solution containing anti-rabbit Alexa 488 secondary antibody diluted 1:1,500, Phalloidin-Tetramethylrhodamine B isothiocyanate (Sigma-Aldrich, P1951) at 1 μg/mL and Hoechst 33258 (Sigma-Aldrich, 94403) at 10 μg/mL was applied to the section for 2 h. After washing 3 × 10 min in PBS slides were mounted using Prolong anti-fade mounting media (ThermoLife Technologies, P10144). Observations were performed with a Zeiss LSM 700 confocal microscope with software Zen 2011 version 7.1. This software was used to determine the distance between bacteria and epithelial cell monolayer, as previously described [21]. In brief, for each animal, 2 high-powered fields (HPFs) were arbitrarily selected with the following inclusion criteria: (1) the presence of stained bacteria; (2) the presence of a clear and delimitated mucosal layer; and (3) the presence of an intact mucus layer. For each HPF, the distance between the 5 closest bacteria and the epithelium was determined. Thus, each bacterial–epithelial distance indicated by a point in the figures is, in fact, the average distance of 10 bacteria–epithelial distances.

### Data presentation and statistical analysis

Data are expressed as means ± SEM and statistical analyses were performed using GraphPad Prism software (V.8.2.0). Significance was determined using one-way analysis of variance (ANOVA), followed by a Bonferroni post hoc test and significant differences were noted as follows: *p ≤ 0.05 **p ≤ 0.01 ***p ≤ 0.001 ****p ≤ 0.0001. For clustering analyzing on principal coordinate plots, categories were compared, and statistical significance of clustering were determined via PERMANOVA.

## Supporting information

S1 Fig Schematic representation of the experimental design used. Mice were exposed to drinking water (blue) containing 1.0% of CMC (orange) or p80 (purple) for 7 weeks, with or without prior weekly immunization with either vehicle (sterile PBS, solid lines and bars) or purified FliC (hatched lines and bars). CMC, carboxymethylcellulose; FliC, flagellin; P80, polysorbate 80.
(TIF)

S2 Fig Dietary emulsifiers consumption modulates the intestinal anti-flagellin IgA response. (**A, B**) Fecal levels of anti-flagellin IgA at week 17, with data being expressed as relative values compared to week 5 nonimmunized group, defined as 100%. The underlying data for this figure can be found in S6 Data. Statistical analyses were performed using a one-way ANOVA and significant differences were recorded as follows: ns: nonsignificant, *p < 0.05, ***p < 0.001, ****p < 0.0001. ANOVA, analysis of variance; CMC, carboxymethylcellulose; FliC, flagellin; IgA, immunoglobulin A; P80, polysorbate 80.
(TIF)

S3 Fig Distribution of IgA index according to relative abundance. Cecal contents were sorted for IgA-positive and IgA-negative bacterial populations. DNA was extracted from sorted cells and subjected to 16S rRNA sequencing, allowing the computing of IgA index for each identified ASVs. Each dot represents identified ASVs, plotted based on their relative abundance and IgA index. (**A**) Water-treated; (**B**) water-treated and flagellin immunized; (**C**) CMC-treated; (**D**) CMC-treated and flagellin immunized; (**E**) P80-treated; (**F**) P80-treated and flagellin immunized. The underlying data for this figure can be found in S7 Data. ASV, amplicon sequence variant; CMC, carboxymethylcellulose; FliC, flagellin; IgA, immunoglobulin A; P80, polysorbate 80.
(TIF)

S4 Fig Microbiota members with a significantly altered IgA index in nonimmunized mice consuming emulsifiers vs. water. Cecal contents were sorted for IgA-positive and IgA-negative bacterial populations at week 19. DNA was extracted from sorted cells and subjected to 16S rRNA sequencing, allowing the computing of an IgA index for each identified ASVs. ASVs with a significantly altered IgA index between immunized and nonimmunized groups were identified and plotted as a heatmap. (**A**) CMC-treated; (**B**) P80-treated. The underlying data for this figure can be found in S8 Data. ASV, amplicon sequence variant; CMC, carboxymethylcellulose; IgA, immunoglobulin A; P80, polysorbate 80.
(TIF)

S5 Fig Microbiota members with a significantly altered IgA index between immunized and nonimmunized mice consuming emulsifiers. Cecal contents, collected at week 19, were sorted for IgA-positive and IgA-negative bacterial populations. DNA was extracted from sorted cells and subjected to 16S rRNA sequencing, allowing computing of IgA index for each identified ASVs. ASVs with a significantly altered IgA index between immunized and non immunized groups were identified and plotted as a heatmap. (**A**) CMC-treated; (**B**) P80-treated. The underlying data for this figure can be found in S9 Data. ASV, amplicon sequence variant; CMC, carboxymethylcellulose; IgA, immunoglobulin A; P80, polysorbate 80.
(TIF)

S1 Data Data used to generate panels 1A, 1B, 1C, and 1D. (XLSX)

S2 Data Data used to generate panel 2D. (XLSX)

S3 Data Data used to generate panels 3A, 3B, and 3C. (XLSX)

S4 Data Data used to generate panels 4A, 4B, 4C, and 4D. (XLSX)

S5 Data Data used to generate panels 5A, 5B, 5C, and 5D. (XLSX)

S6 Data Data used to generate panels S2A and S2B. (XLSX)

S7 Data Data used to generate panels S3A, S3B, S3C, S3D, S3E, and S2F. (XLSX)

S8 Data Data used to generate panels S4A and S4B. (XLSX)

S9 Data Data used to generate panels S5A and S5B. (XLSX)

## Abbreviations

ANOVA: analysis of variance
CMC: carboxymethylcellulose
FISH: fluorescent in situ hybridization
HE: hematoxylin–eosin
HEK: human embryonic kidney
HPF: high-powered field
HPLC: high-performance liquid chromatography
IBD: inflammatory bowel disease
Ig: immunoglobulin
IgA: immunoglobulin A
IgG: immunoglobulin G
Lcn2: lipocalin 2
LGI: low-grade inflammation
LPS: lipopolysaccharide
PCoA: principal coordinate analysis
NRS: normal rat serum
P80: polysorbate 80
qRT-PCR: quantitative reverse transcription PCR
SC/FSC: side scattered light/forward scatter
TLR: Toll-like receptor
WT: wild-type
