# Supplementary figures and images for: Vaccination against microbiota motility protects mice from the detrimental impact of dietary emulsifier consumption
Source: PLoS Biol. 2023 Sep 19;21(9):e3002289. doi: 10.1371/journal.pbio.3002289 (PMC10508614; doi:10.1371/journal.pbio.3002289)

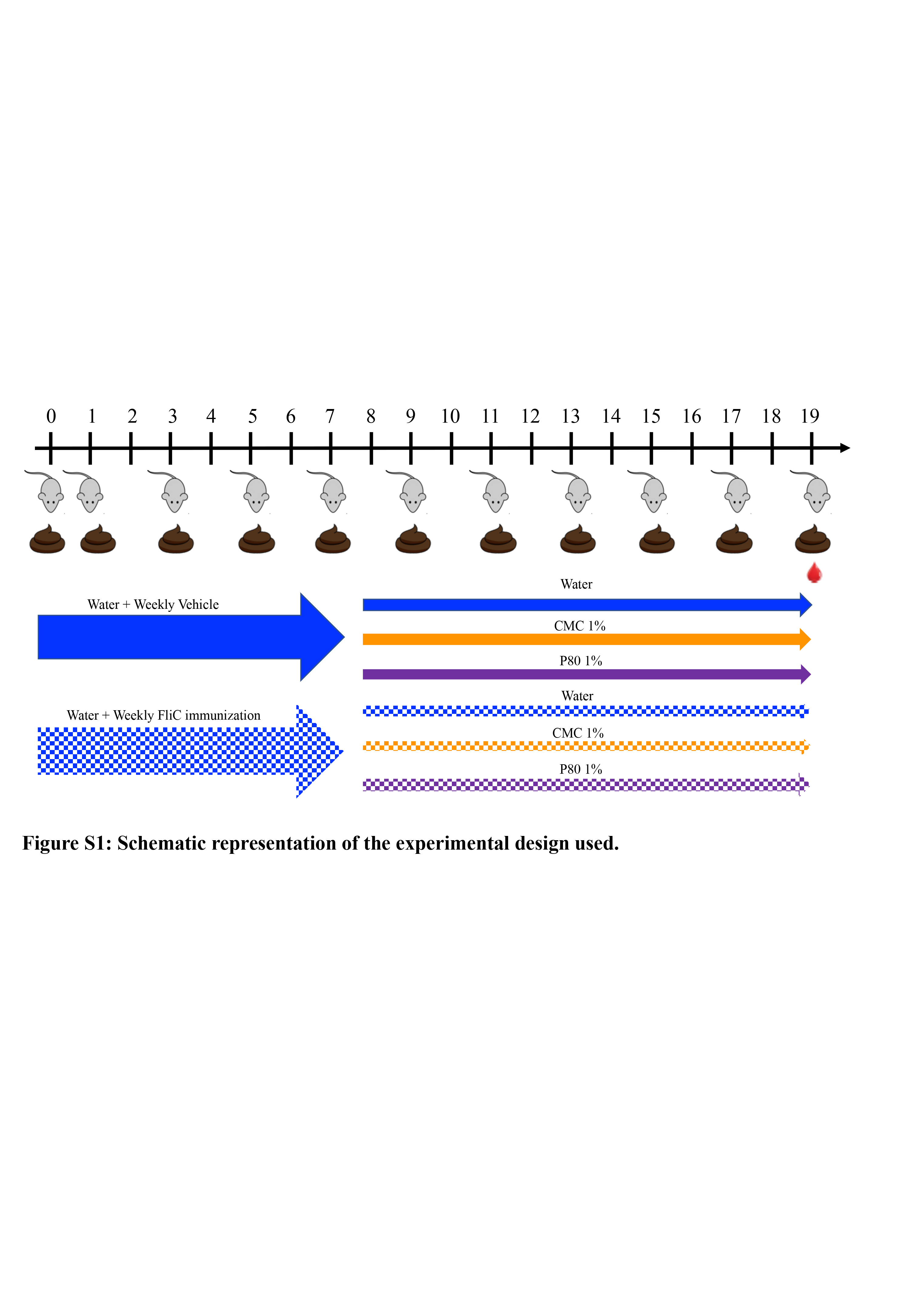

Supplement: S1 Fig — Mice were exposed to drinking water (blue) containing 1.0% of CMC (orange) or p80 (purple) for 7 weeks, with or without prior weekly immunization with either vehicle (sterile PBS, solid lines and bars) or purified FliC (hatched lines and bars). CMC, carboxymethylcellulose; FliC, flagellin; P80, polysorbate 80. (TIF) [file pbio.3002289.s001.tif]

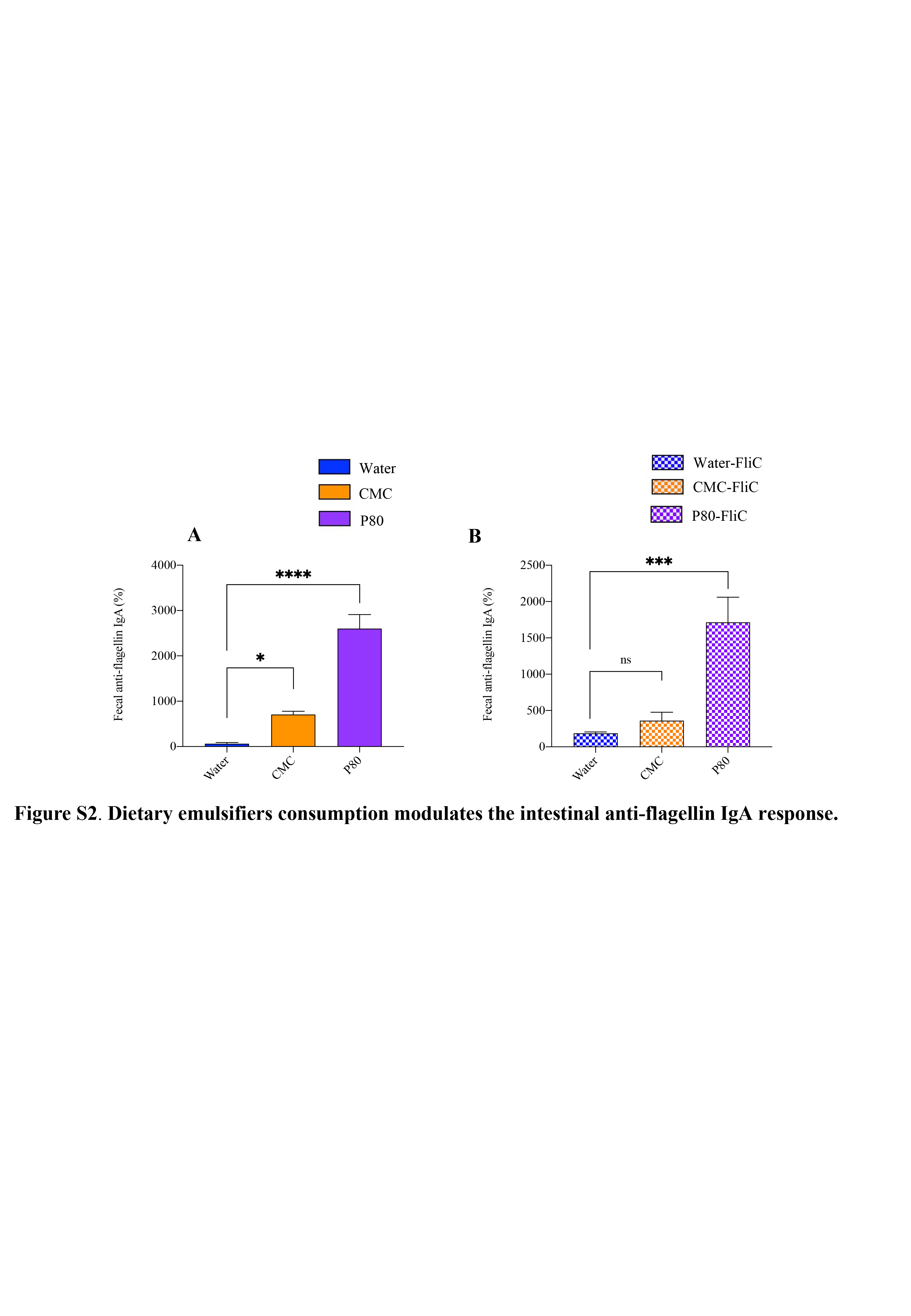

Supplement: S2 Fig — (A, B) Fecal levels of anti-flagellin IgA at week 17, with data being expressed as relative values compared to week 5 nonimmunized group, defined as 100%. The underlying data for this figure can be found in S6 Data. Statistical analyses were performed using a one-way ANOVA and significant differences were recorded as follows: ns: nonsignificant, *p < 0.05, ***p < 0.001, ****p < 0.0001. ANOVA, analysis of variance; CMC, carboxymethylcellulose; FliC, flagellin; IgA, immunoglobulin A; P80, polysorbate 80. (TIF) [file pbio.3002289.s002.tif]

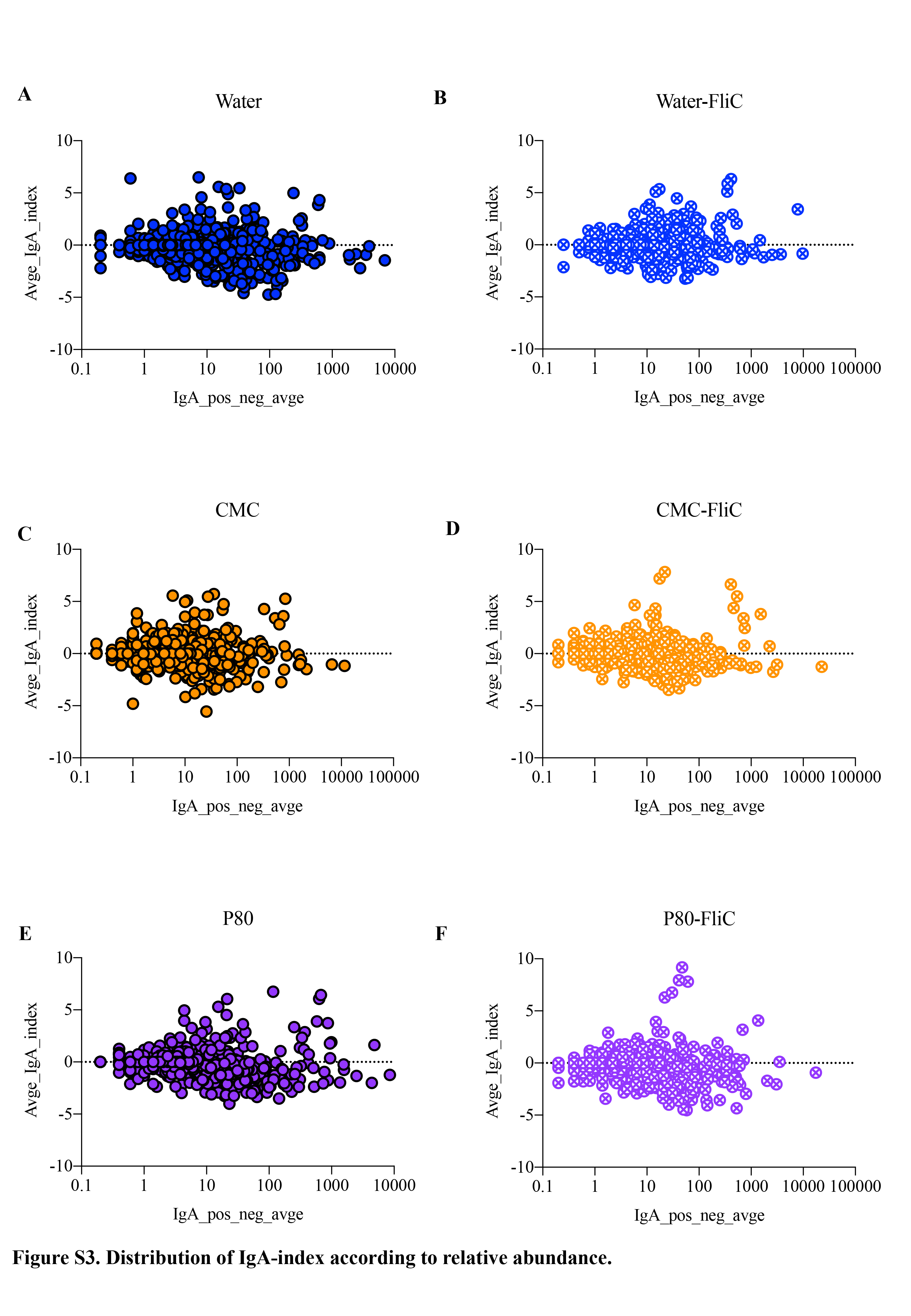

Supplement: S3 Fig — Cecal contents were sorted for IgA-positive and IgA-negative bacterial populations. DNA was extracted from sorted cells and subjected to 16S rRNA sequencing, allowing the computing of IgA index for each identified ASVs. Each dot represents identified ASVs, plotted based on their relative abundance and IgA index. (A) Water-treated; (B) water-treated and flagellin immunized; (C) CMC-treated; (D) CMC-treated and flagellin immunized; (E) P80-treated; (F) P80-treated and flagellin immunized. The underlying data for this figure can be found in S7 Data. ASV, amplicon sequence variant; CMC, carboxymethylcellulose; FliC, flagellin; IgA, immunoglobulin A; P80, polysorbate 80. (TIF) [file pbio.3002289.s003.tif]

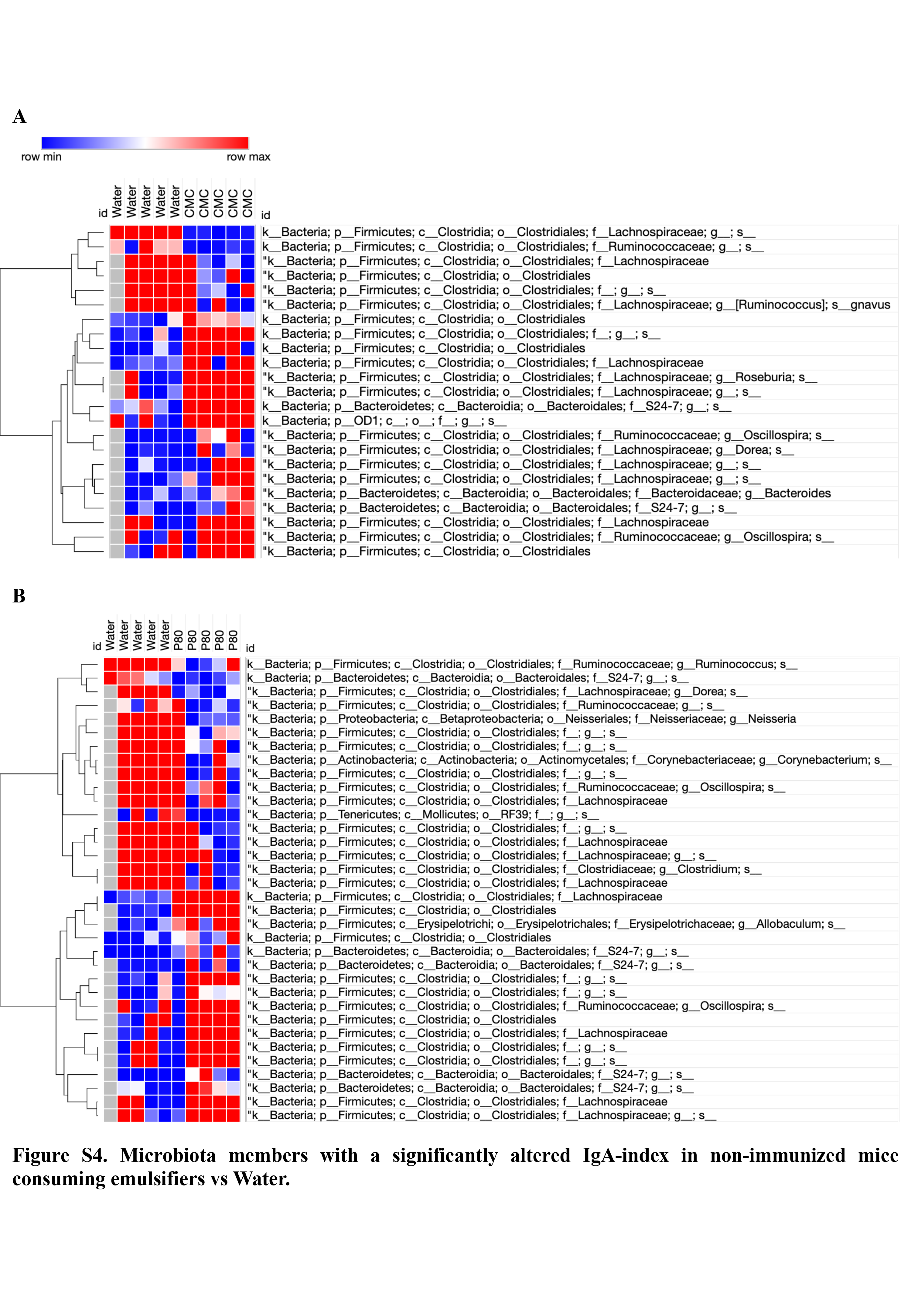

Supplement: S4 Fig — Cecal contents were sorted for IgA-positive and IgA-negative bacterial populations at week 19. DNA was extracted from sorted cells and subjected to 16S rRNA sequencing, allowing the computing of an IgA index for each identified ASVs. ASVs with a significantly altered IgA index between immunized and nonimmunized groups were identified and plotted as a heatmap. (A) CMC-treated; (B) P80-treated. The underlying data for this figure can be found in S8 Data. ASV, amplicon sequence variant; CMC, carboxymethylcellulose; IgA, immunoglobulin A; P80, polysorbate 80. (TIF) [file pbio.3002289.s004.tif]

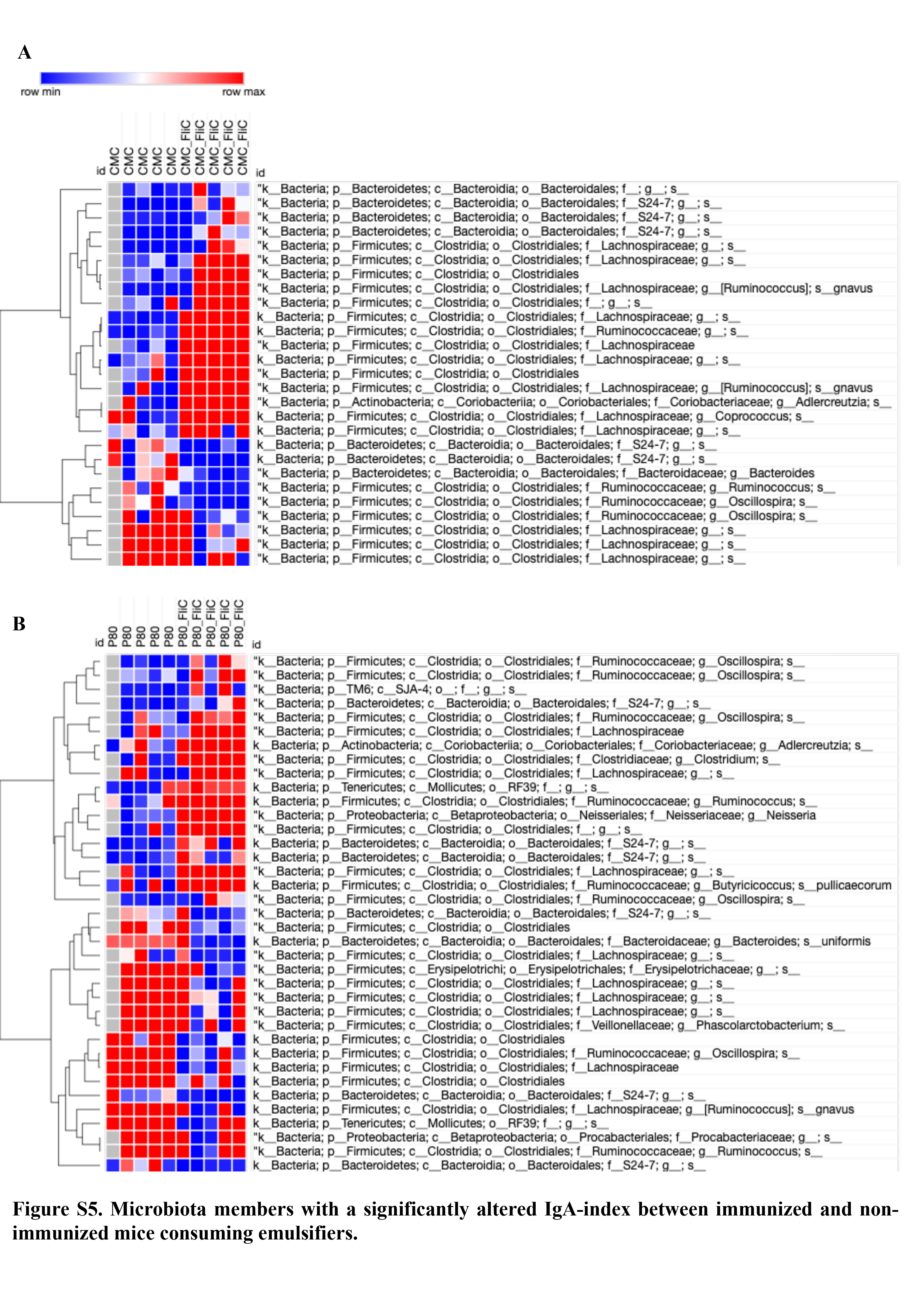

Supplement: S5 Fig — Cecal contents, collected at week 19, were sorted for IgA-positive and IgA-negative bacterial populations. DNA was extracted from sorted cells and subjected to 16S rRNA sequencing, allowing computing of IgA index for each identified ASVs. ASVs with a significantly altered IgA index between immunized and non immunized groups were identified and plotted as a heatmap. (A) CMC-treated; (B) P80-treated. The underlying data for this figure can be found in S9 Data. ASV, amplicon sequence variant; CMC, carboxymethylcellulose; IgA, immunoglobulin A; P80, polysorbate 80. (TIF) [file pbio.3002289.s005.tif]
